# *Salmonella* Serotyping; Comparison of the Traditional Method to a Microarray-Based Method and an *in silico* Platform Using Whole Genome Sequencing Data

**DOI:** 10.3389/fmicb.2019.02554

**Published:** 2019-11-11

**Authors:** Benjamin Diep, Caroline Barretto, Anne-Catherine Portmann, Coralie Fournier, Aneta Karczmarek, Guido Voets, Shaoting Li, Xiangyu Deng, Adrianne Klijn

**Affiliations:** ^1^Nestlé Research, Lausanne, Switzerland; ^2^Check-Points, Wageningen, Netherlands; ^3^Center for Food Safety, University of Georgia, Athens, GA, United States

**Keywords:** *Salmonella* serotyping, microarray, WGS, phenotypic method, methods comparison, *Salmonella enterica*

## Abstract

*Salmonella* is one of the most common causes of food-borne diseases worldwide. While *Salmonella* molecular subtyping by Whole Genome Sequencing (WGS) is increasingly used for outbreak and source tracking investigations, serotyping remains as a first-line characterization of *Salmonella* isolates. The traditional phenotypic method for serotyping is logistically challenging, as it requires the use of more than 150 specific antisera and well trained personnel to interpret the results. Consequently, it is not a routine method for the majority of laboratories. Several rapid molecular methods targeting O and H loci or surrogate genomic markers have been developed as alternative solutions. With the expansion of WGS, *in silico Salmonella* serotype prediction using WGS data is available. Here, we compared a microarray method using molecular markers, the Check and Trace *Salmonella* assay (CTS) and a WGS-based serotype prediction tool that targets molecular determinants of serotype (SeqSero) to the traditional phenotypic method using 100 strains representing 45 common and uncommon serotypes. Compared to the traditional method, the CTS assay correctly serotyped 97% of the strains, four strains gave a double serotype prediction. Among the inconclusive data, one strain was not predicted and two strains were incorrectly identified. SeqSero was evaluated with two versions (SeqSero 1 and the alpha test version of SeqSero 2). The correct antigenic formula was predicted by SeqSero 1 for 96 and 95% of strains using raw reads and assembly, respectively. However, 34 and 33% of these predictions included multiple serotypes by raw reads and assembly. With raw reads, one strain was not identified and three strains were discordant with phenotypic serotyping result. With assembly, three strains were not predicted and two strains were incorrectly predicted. While still under development, SeqSero 2 maintained the accuracy of antigenic formula prediction at 98% and reduced multiple serotype prediction rate to 13%. One strain had no prediction and one strain was incorrectly predicted. Our study indicates that the CTS assay is a good alternative for routine laboratories as it is an easy to use method with a short turn-around-time. SeqSero is a reliable replacement for phenotypic serotyping if WGS is routinely implemented.

## Introduction

*Salmonella* is one of the most common causes of food-borne diseases worldwide and is a public health concern in both industrialized and developing countries (Scallan et al., 2011; Oh and Park, 2017). Non-typhoïd salmonellosis represents the majority of *Salmonella* infections in humans. It results in tens of millions of human infections globally each year^1^. In the EU, 100 000 cases are reported each year with an estimated cost of €3 billion a year^2^. In the United States, the number of cases goes up to 1 million per year causing an annual loss of $3.3 billion USD. This number includes the destruction of contaminated food commodities, loss of work productivity, and health-care costs (Hoffmann et al., 2012). In order to reduce the number of *Salmonella* cases, national authorities are emphasizing the need to control *Salmonella* along the food chain, from primary production to consumption via surveillance program in accordance with local legislation (e.g., EU legislation EC 2016/2003). Food-borne outbreaks are epidemiologically investigated and reported (Hugas and Beloeil, 2014). Food safety management systems including good manufacturing practices are put in place to ensure food safety. *Salmonella* detection is tested by a reference method (ISO 6579, 2017) or validated alternative methods. Once a presumptive *Salmonella* is detected, the isolate must be confirmed and often the serotype is identified. Serotyping remains the first step to characterize *Salmonella* isolates although it does not provide sufficient discriminatory subtyping for outbreaks investigation. The traditional method to determine a *Salmonella* serotype is a phenotypic method, based on the WKL scheme (Grimont and Weill, 2007). The serotype is determined by agglutination of the bacteria with specific antisera to identify variants of somatic (O) and flagella (H) antigens. This provides the antigenic formula of the strain associated to the name and subspecies of the serotype. To date, 46 O antigens and 114 H antigens are identified that, in various combinations, characterize more than 2600 reported serotypes (Issenhuth-Jeanjean et al., 2014). The drawback of the traditional phenotypic method is that it requires the availability of more than 150 specific antisera and well-trained personnel to correctly interpret the results (Wattiau et al., 2011). Consequently, it is not possible for all laboratories to carry out this method in-house, and often, laboratories have to send the isolates to a national reference laboratory, an expert laboratory or a commercial laboratory. This process can significantly delay the time to result. In terms of performance, the method may give false positive reactions due to weak or non-specific agglutination. Autoagglutination or loss of antigen expression, as observed for rough and non-motile isolates, results in unidentified serotypes (Wattiau et al., 2011). Numerous alternative methods have been developed (Wattiau et al., 2011). These methods include (i) serotyping based on O and H antigen loci using PCR-based methods (Herrera-Leon et al., 2007) and microarray-based methods (McQuiston et al., 2011), (ii) serotyping based on surrogate genomic markers such as virulence genes (Peterson et al., 2010). With the advent of NGS and the decrease in sequencing cost, WGS is becoming increasingly more affordable and represents a powerful tool for pathogen subtyping, source tracking and characterization such as virulence and antimicrobial resistance gene profiling (Deng et al., 2016; Rantsiou et al., 2018). Since serotyping remains the first step in *Salmonella* characterization, several *in silico* platforms utilizing WGS data to predict the serotypes have been developed (Zhang et al., 2015; Asthon et al., 2016; Yoshida et al., 2016) and evaluated by public health agencies (Yachison et al., 2017). While WGS is increasingly used in routine by public health and regulatory agencies and authorities (e.g., the U.S. Food & Drug Administration (FDA), Public Health England), food industry has only recently started exploring WGS (Rantsiou et al., 2018; Rouzeau-Szynalski et al., 2019). The use of WGS and its application including *Salmonella* serotyping in routine laboratories are becoming a viable option. In our study, we compared the traditional phenotypic method to the CTS assay, a proprietary method commercialized by Check-Points (Netherlands) and *in silico* platform SeqSero. The CTS assay, formerly Premi^®^Test, has been available since 2007 (Wattiau et al., 2008). It provides a fast and easy-to-use platform for *Salmonella enterica* subsp. *enterica* serotyping and showing good concordance with the traditional phenotypic method (Wattiau et al., 2008; Ferrato et al., 2017). The method is also successfully validated by the AOAC for more than 100 serotypes and by the OIE for more than 20 serotypes. This makes the CTS assay a good candidate to implement in an ISO17025 accredited laboratory. The latest version of the method also includes O and H gene markers and the current database contains patterns for over 300 serotypes (personal communication). SeqSero mainly targets genetic determinants of O and H antigens, including the *fliC* and *fljB* genes and the *wzx* or *wzy* genes in the *rfb* region (Zhang et al., 2015). This is the first study that compared the traditional serotyping using antisera to a rapid proprietary molecular method (CTS) and a WGS-based serotyping by serotype determinants (SeqSero) with the same set of 100 *Salmonella* strains covering 45 serotypes.

## Materials and Methods

### Selection of *Salmonella* Serotypes

One hundred *Salmonella enterica* subsp. *enterica* strains were selected, covering the most common serotypes encountered in the United States and Europe, supplemented with strains representing a variety of O antigen groups. Only *Salmonella enterica* subsp. *enterica* were selected due to the scope of the CTS assay. *S. Typhimurium* monophasic variants and its close antigenic formula related serotypes (*S.* Lagos, *S.* Agama) were also included to challenge the methods. Where possible, each serotype was tested with three different strains (Table 1). The strains were obtained from the EURL for *Salmonella* (EURL-*Salmonella*), strain collection, located at the (RIVM, Bilthoven, Netherlands). All strains previously have been part of the EURL-*Salmonella* Proficiency Testing schemes for serotyping, and originated from human, food or environmental sources^3^. Duplicate sets of blindly coded strains were sent to Nestlé Research (Lausanne, Switzerland) for sequencing and *in silico* testing, and to Check-Points (Check-Points B.V., Wageningen, Netherlands) for the CTS testing.

**TABLE 1:** Strains used in this study.

**Top common serotypes**	***S. Typhimurium*, its monophasic variants and close antigenic formula serotypes**	**Other serotypes**
Enteritidis^a b^ (3)	Typhimurium (3)^∗^	O4: Arechavaleta (1), Heidelberg (3), Indiana (3), Kiambu (1)
Typhimurium^a b^ (3)^∗^	1,4,[5]:12:i (3)^∗^	O7: Cholearesuis (1), Mbandaka (3), Montevideo (3), Ohio (1), Oranienburg (3), Tennessee (3)
Newport^a b^ (3)	Lagos (1)	O8: Albany (3), Goldcoast (2),Hadar (3), Molade (2)
1,4,[5]:12:i^a b^ (3)^∗^	Agama (3)	O9: Dublin (3), Miami (1), Panama (1)
Muenchen^a^ (2)		O9, 46: Ouakam (1)
Infantis^a b^ (3)		O3,10: Give (3)
Braenderup^a^ (3)		O1,3,19: Ahmadi (1), Liverpool (2), Senftenberg (3)
Saintpaul^a^ (2)		O11: Abaetetuba (1)
Thompson^a^ (3)		O13: Bracknell (3), Putten (1), Poona (3)
Virchow^b^ (3)		O16: Yoruba (3)
Agona^b^ (2)		O17: Jangwani (1)
Kentucky^b^ (3)		O21: Ruiru (1)
		O30: Urbana (1)
		O35: Adelaide (2)

*^*a*^CDC (2017) https://www.cdc.gov/foodnet/reports/prelim-data-2017.html. ^*b*^EFSA (2017) http://www.efsa.europa.eu/sites/default/files/scientific_output/efs2_5500_Rev2.pdf. ( ): number of strains. ( )^∗^: same strains.*

### Traditional Phenotypic Serotyping

Strains were originally received on agar transport tubes and purified on blood agar before actual serotyping. The antigenic formula of the strains was determined by the EURL-*Salmonella* using standard agglutination methods (ISO/TR 6579-3, 2014), and the serotype name was assigned according to the WKL scheme (Grimont and Weill, 2007). Identification of a monophasic variant of *S. Typhimurium* was confirmed by PCR (Tennant et al., 2010).

### CTS Assay

After the *Salmonella* strains were streaked on TSA plates and incubated o/n at 37°C ± 1°C, lysis was performed by taking one colony and adding it to 100 μl Lysis Buffer which was subsequently placed at 98°C for 5 min. The CTS array uses multiplex LDRs to generate circular DNA molecules from the probes, which are amplified in a subsequent step using a single primer pair (Schouten et al., 2002; van Eijk et al., 2004). After amplification, the PCR products are hybridized to a specific location on the microarray due to the presence of an unique DNA sequence, or zip, incorporated in the PCR primer that is complementary to a sequence on the microarray. The proprietary genomic markers that were amplified then become visible on unique locations, creating a microarray hybridization profile that can identify and discriminate between different *S. enterica* subsp. *enterica* serotypes (Wattiau et al., 2008). The proprietary software of Check-Points B.V. assigns a numeric code named genovar code to each pattern on the microarray. Subsequently, the software compares the genovar code to a database and provides the end-user with a *S. enterica* subsp. *enterica* serotype.

#### Ligation, Exonuclease and Amplification

*Salmonella* strains were processed as described in version 9.2 of the user manual of the CTS test, except the following: lysis was performed for 5 min. A freshly prepared proprietary mix containing ligation probes and thermostable DNA ligase was added to 10 μl of crude DNA extract. Ligation was performed using the following protocol: step (1) 3 min at 95°C, step (2) 25 cycles of 30 s at 95°C and 4 min at 65°C and step (3) 2 min at 95°C. The incubation of the exonuclease was performed for 30 min. Probe amplification was performed using the following protocol: step (1) 10 min at 95°C, step (2) 30 cycles of 30 s at 95°C, 30 s at 55°, and 60 s at 72°C, and step (3) 2 min at 95°C. These modifications are the updated protocol, designed to reduce overall time-to-result without eliminating steps or altering the chemical reactions.

#### DNA Hybridization, Conjugation, and Detection

*Salmonella* strains were processed as described in version 9.2 of the user manual of the CTS test, except the following: all incubations were done for 1 min, blocking was done for 3 min and 5 min, respectively, and finally conjugation was done for 12 min.

#### Analysis

Computer analysis was performed as described in the user manual (version 9.2) using the Check-Points Tube Reader and the proprietary computer software developed by Check-Points B.V. (Check-Points B.V., Wageningen, Netherlands).

### Serotype Prediction by SeqSero

#### Strain Purification and Preparation

*Salmonella* strains were first streaked on XLD plate and incubated at 37°C ± 1°C for 24 h ± 2 h for confirmation. Typical colonies were streaked on TSA plates and incubated at 37°C ± 1°C for 18–24 h. One isolated colony was subsequently cultured in 4 mL of pre-heated BHI broth at 37°C ± 1°C for 5–8 h. After incubation, 1 ml of BHI broth was transferred into an Eppendorf tube and centrifuged at 5000 *g* for 5 min. Supernatant was then discarded and the pellet was collected for DNA extraction.

#### DNA Extraction

DNA extraction was performed using the Maxwell RSC system version 2. Bacterial pellet was first resuspended in 160 μL of buffer P1 (Qiagen 19051), mixed by vortexing then spun down. Twenty μL of freshly prepared lysozyme (Sigma L6876-1g) were added and incubated at 37°C shaking at 600 rpm for 30 min. Forty μL of RNase A (Sigma-Aldrich 4873) were added and left at room temperature for 2 min. The total volume (220 μL) was transferred into the RCS cartridge kit and followed the manual instruction.

#### DNA Short Read Sequencing

DNA was normalized at 0.2 ng/μL in order to start with 1 ng to perform a sequencing library preparation using Nextera XT kit (Illumina) following the supplier’s instructions. A final AMPure beads purification at ratio 0.6 was performed on a Sciclone robotic platform from Perkin Elmer. The quality and quantity of each library were evaluated using a capillary electrophoresis method (LabChip GX Touch from Perkin Elmer). Libraries were pooled based on molarity calculated by the LabChip GX Touch. The equimolar pool was assembled using a Hamilton robotic platform. To ensure each library was present in the pool before sequencing, the equimolar pool was controlled by a MiSeq run v2 chemistry for 2 × 20 cycles. The pool of 188 samples including positive and negative controls, was sequenced on a HiSeq 2500 platform (Illumina) using Rapid v2 chemistry in PE250. The pool was spiked with 2% PhiX, loaded at 8pM on one flow cell.

#### SeqSero

##### Raw data quality check

The FastQC software (v0.11.5) was used to evaluate the quality of raw sequencing reads according to the FasQC threshold^4^.

##### In silico serotype prediction

SeqSero analysis was performed using two versions of the software: published SeqSero 1^5^ and the alpha test version SeqSero 2^6^ from September 2018 for which new algorithms were implemented. SeqSero 1 and SeqSero 2 allow serotyping prediction from raw reads and genome assemblies. Both types of data for all the strains used in this study were tested with SeqSero 1 and SeqSero 2. In addition, SeqSero 2 provides a k-mer based algorithm which allows rapid serotype prediction from raw reads (seconds per genome) and improves serotype prediction from draft genome assemblies. The allele microassembly workflow performs targeted assemblies of serotype determinant genes, instead of assembling the entire genomes for serotype prediction.

##### De novo assembly

Trimmomatic v.0.36 (Bolger et al., 2014) was used to trim and filter low quality reads. It was used with the options PE -phred33 leading: 20 trailing:20 sliding window:10:20 minlen:150. SKESA v.2.2 software was used with –use_paired_ends, – vector_percent 1 and – allow_snps parameters to generate all *de novo* assembly (Souvorov et al., 2018). If a draft genome assembled by SKESA resulted in no/incomplete prediction, the genome was re-assembled by SPAdes (Bankevich et al., 2012) and re-analyzed by SeqSero 1 and SeqSero 2.

## Results

### CTS Assay Results

A total of 100 *Salmonella enterica* subsp. *enterica* strains representing 45 different serotypes covering the most common serotypes encountered in human infection, *S. Typhimurium* and its monophasic variant and different O groups were included in this study. Overall results are presented in Table 2. Full agreement indicates that the result obtained with the alternative method was 100% concordant with the traditional phenotypic method. Multiple prediction indicates that the alternative method proposed several serotype predictions, which included the serotype determined by the traditional phenotypic method. No/incomplete prediction indicates that the alternative method was not able to provide a serotype. In the case of CTS assay, a genovar code is assigned. Disagreement indicates that the result obtained with the alternative method is different from the traditional phenotypic method. All CTS results were obtained with a novel protocol, designed to reduce overall time-to-result without eliminating steps or altering the chemical reactions. Ninety three out of 100 strains tested with the CTS assay were assigned with a unique serotype, were concordant with the traditional phenotypic method results. Four out of 100 strains (PIR02262, PIR02284, PIR02297, and PIR02323) were given a double identification. Two disagreement (PIR02268: *S.* Brandenburg instead of *S.* Poona, PIR02337: *S. Typhimurium* instead of 1,4,[5],12:i:-) were observed. One strain (PIR02242) was assigned by a unique genovar code (genovar code 7213 instead of *S.* Adelaide), which was not correlated with a serotype in the CTS database (Table 3). For all the most prevalent serotypes, CTS correctly assigned the serotype. *S. Typhimurium* and other O:4,H:i strains such as *S.* Agama (*n* = 3), *S.* Lagos (*n* = 1) were also correctly identified by CTS. For the *S. Typhimurium* monophasic variants (1,4,[5],12:i:- (*n* = 3), two were correctly identified as monophasic serotype but one (PIR02337) was identified as *S. Typhimurium*.

**TABLE 2:** Overall results of CTS and SeqSero compared to traditional phenotypic method.

		**Full**	**Multiple**	**No/incomplete**	
**Strains**	**Method**	**agreement**	**prediction^a^**	**prediction^b^**	**Disagreement**
EURL-*Salmonella*	CTS	93%	4%	1%	2%
strains (100 strains)	SeqSero 1 raw reads	62%	34%	1%	3%
	SeqSero 1 assembly	62%	33%	3%	2%
	SeqSero 2 raw reads allele microassembly	85%	13%	1%	1%
	SeqSero 2 raw reads kmer	85%	13%	1%	1%
	SeqSero 2 assembly	85%	13%	1%	1%

*^*a*^Multiple predictions includes the expected serotype. ^*b*^For SeqSero, No/incomplete prediction includes an incomplete antigenic formula.*

**TABLE 3:** Check and trace *Salmonella* assay discrepant results.

	**Traditional**		
**Strain**	**phenotypic**		
**number**	**method**	**CTS results**	**Antigenic formula**
PIR02242	Adelaide	genovar 7213	Adelaide: 35:f,g: -:[z_27_]
PIR02262	Arechavaleta	Arechavaleta or Kisangani	Arechavaleta: 4,[5],12:a:1,7
			Kisangani: 1,4,[5],12:a:1,2
PIR02268	Poona	Brandenburg	Poona: 1,13,22:z:1,6:[z_44_], [z_59_]
			Brandenburg: 4,[15],12:i,v:e,n,z_15_
PIR02284	Bracknell	Bracknell or	Bracknell: 13,23:b:1,6
		Oudwijk	Oudwijk: 13,22:b:1,6
PIR02297	Bracknell	Bracknell or	Bracknell: 13,23:b:1,6
		Oudwijk	Oudwijk: 13,22:b:1,6
PIR02323	Bracknell	Bracknell or	Bracknell: 13,23:b:1,6
		Oudwijk	Oudwijk: 13,22:b:1,6
PIR02337	1,4,5,12:i:-	Typhimurium	

### SeqSero Results

Raw sequence data passed the FastQC thresholds with R1 and R2 flagged “PASS” with the exception of one sample shown as “WARN” of R2, indicating an overall high quality of sequences. The overall results suggested that SeqSero 2 (alpha test version) improved serotype prediction compared to SeqSero 1. Predictions in full agreement with traditional serotyping were increased from 62% (raw reads, assembly) to 85% (raw reads and assembly). Multiple predictions, which included the correct serotype, were reduced from 33% (assembly) and 34% (raw reads) to 13% (raw reads, assembly) with SeqSero 2. Predictions in disagreement with traditional method were reduced from 3% (raw reads) and 2% (assembly) to 1% (raw reads, assembly) (Table 2). The occurrences of no/incomplete predictions were reduced from 3% to 1% (assembly) or remained at 1% (raw reads). Supplementary Table S2 shows the details of the discrepant results. For SeqSero 1, 34 strains displayed multiple predictions when using raw reads. This number was 33 when using assembly. However, the number of no/incomplete prediction was higher with the assembly mode. A total of three strains [PIR02287 (*S*. Mbandaka), PIR02263 (*S*. Ruiru), and PIR02315 (*S*. Wirchow)] were not serotyped using assembly and only one strain [PIR02261 (*S*. Ouakam)] was not serotyped by SeqSero 1 using raw reads. SeqSero 1 raw reads mode incorrectly predicted the serotype of three strains [PIR02295 (*S*. Lagos), PIR02330 (*S*. Enteritis) and PIR02337 (1,4,[5],12:i:- identified as *S. Typhimurium*] and the assembly mode incorrectly predicted two strains [PIR02303 (*S. Choleraesuis*) and PIR02294 (*S*. Thompson)]. The alpha test version of SeqSero 2 was also evaluated with the same set of strains. Raw reads were analyzed using both allele microassembly and K-mer modes. Identical predictions were obtained by the two modes. One 1,4,5,12:i:- strain [PIR02337 (monophasic variant of Typhimrium)] was predicted to be Typhimurium. A serotype Ouakam strain (PIR02261) was not identified because of incomplete prediction of antigenic formula. Using assembly, same results were observed; no/incomplete prediction for strain PIR02261 (*S.* Ouakam) and incorrect prediction for strain PIR02337 [*S. Typhimurim* instead of 1,4,[5],12:i:-]. Interestingly, antigenic formulae of strains PIR02239 (*S*. Tennessee) and PIR02294 (*S*. Thompson) were correctly predicted when their genomes were assembled by SPAdes, but incompletely (not full antigenic formula was displayed) predicted if their genomes were assembled by SKESA. This difference comes from the conservative algorithm used by SKESA creating less contiguous assemblies than those generated by other assemblers such as SPAdes (Souvorov et al., 2018).

## Discussion

*Salmonella* serotyping remains the first step of *Salmonella* characterization in surveillance programs, source tracking and epidemiological investigation. Molecular methods have been used as alternatives to traditional phenotypic serotyping method. In this study, a set of 100 blind coded strains was prepared by the EURL-*Salmonella*, and sent to Nestle Research and Check-Points for testing by SeqSero and CTS assay, respectively. Supplementary Table S1 shows the details of all results. The CTS assay is a good alternative to phenotypic serotyping with 93% of the results in full agreement. These results concur with previous studies (Wattiau et al., 2008; Ferrato et al., 2017). The CTS assay was able to identify most of the common *Salmonella* serotypes tested, including *S. Typhimurium* and its monophasic variants. However, one monophasic variant was identified as *S. Typhimurim*. Bugarel et al. (2012) has described genetic variation among *S. Typhimurium* monophasic strains with 15% of them containing all the genomic markers of a *S. Typhimurium*. Since the CTS assay mainly relies on genomic markers, there is a possibility that this specific strain resembles *S. Typhimurium* genetically, but phenotypically matches the monophasic variant. This study also shows that the CTS assay correctly identified *S*. Agama and *S*. Lagos. Both results are an improvement in comparison to the study of Madajczak (Madajczak et al., 2015) which showed that CTS assay correctly identified most of the *S. Typhimurium* and its monophasic variants but failed to recognize *S*. Agama and *S*. Lagos. This improvement is linked to the continuous evolution of the database. New serotypes are added if strains from that serotype have been obtained from three different locations and the serotype has been confirmed by a reference laboratory using the traditional phenotypic method. At the time of this study, the CTS assay has been validated and approved for more than 130 serotypes by AOAC and more than 20 serotypes by OIE. The performance of the CTS assay has been also demonstrated by comparing to the traditional phenotypic method (Wattiau et al., 2008; Ferrato et al., 2017) and molecular methods targeting specific genomic markers or O and H antigen determinants (Beaubrun et al., 2014). In this study, two serotypes obtained a double prediction with CTS assay (*S.* Arechavaleta and *S.* Bracknell). Such a double prediction is not unexpected as the proprietary molecular markers of the CTS assay are mainly surrogate markers, which can lead to difficulties separating closely related serotypes. Additional genomic markers will increase the specificity. In other situations, the CTS assay provided more than one genovar pattern for one serotype. The patterns within one serotype can differ due to the presence/absence of one or more genomic marker(s). This was observed with one strain (PIR02242) which, provided a new genovar code (genovar 7213) for a serotype that was known to the CTS database (*S*. Adelaide). While different patterns for one serotype suggest that the CTS assay can go beyond serotyping, it is not sufficient for strain subtyping. SeqSero 1 and SeqSero 2 (alpha test version), in theory, are able to predict more than 2200 serotypes, of which 45 were tested in this study (Zhang et al., 2015). Two incorrect predictions were made using the raw reads mode of SeqSero 1. These were due to incorrect classification of H antigen alleles, including a serotype Enteritidis strain predicted as Blegdam and a Lagos strain predicted as Tsevie. Both strains were correctly serotyped by SeqSero 2 with all modes. A serotype 1,4,5,12:1:- strain (monophasic variant of Typhimurium) was predicted to be Typhimurium by all modes of SeqSero 1 and SeqSero 2 except the assembly mode of SeqSero 1. This strain was found to be genotypically diphasic by carrying a *fljB* allele. It is possible that this gene was not expressed in the strain (e.g., a pseudogene), leading to the monophasic determination by the phenotypic method. The monophasic prediction by the assembly mode of SeqSero 1 was due to unsuccessful extraction of the *fljB* allele from the genome assembly using *in silico* PCR, which is unique to SeqSero 1, sensitive to assembly quality, and no longer used by SeqSero 2. A serotype Ouakam strain was only predicted by the assembly mode of SeqSero 1. The other modes of SeqSero 1 and SeqSero 2 generated incorrect O antigen prediction (9 instead of 9, 46). The prediction of O group 9, 46 requires identification of *wzy* and *wbaV* alleles. The *wzy* allele sequence in the WGS of this strain was truncated, suggesting either a rare allele of this gene or incomplete sequencing of the gene. The genome assembly mode assigned the correct serotype because it uses the entire *rfb* gene cluster from the genome assembly for O antigen identification. SeqSero 2 was still under development at the time of this study. The version tested in this study showed improved performance over SeqSero 1 as there was only one misidentification (*S. Typhimurium* monophasic variant). The release version of the SeqSero 2 software was recently published (Zhang et al., 2019) but was not included in this study. SeqSero 1 is accurate to a large extent at predicting antigenic formulae of *Salmonella* serotypes consistent with traditional phenotypic serotyping of *Salmonella*. This feature provides important continuity with historical surveillance and research data based on *Salmonella* serotypes. However, the interpretation of SeqSero 1 predicted antigenic formulae was confounded by multiple factors, which sometimes led to indefinitive serotype predictions. First, the same antigenic formula can be shared by strains from different subspecies, such as Oranienburg shared with subspecies II, II 6,7:m,t:-. SeqSero 1 was not able to identify *Salmonella* subspecies and assign all the possible serotype names associated with a formula. Second, some serotypes in the WKL scheme require additional phenotypes for differentiation such as Paratyphi B and Paratyphi B var. L (+) tartrate (formerly known as Java). Differentiation of serotypes or serotype variants like these require additional markers, which ideally are genetic determinants of the differentiating phenotypes. Third, some serotypes in the WKL scheme differ only by minor epitopes of the same O antigen group. Markers for these epitopes were often not available in SeqSero 1. Our evaluation study indicates that some of these issues have been addressed in SeqSero 2. While still an alpha test version, SeqSero 2 correctly distinguished all the strains that were indefinitely predicted by SeqSero 1 due to lack of subspecies identification. Our study confirms that methods based on molecular genomic markers (CTS) or specifically H and O markers (SeqSero) can be used for *Salmonella* serotyping. For laboratories without routine access to WGS, commercialized molecular methods such as the CTS assay are a good alternative. Indeed, molecular methods have been intensively implemented in routine laboratories the last 10 years. The CTS assay results are obtained within 8 h from the isolated colony. The CTS assay is an easy to use method provided basic molecular techniques and laboratory good practices are strictly applied (Ferrato et al., 2017). In comparison, this is much faster than traditional phenotypic method, which usually takes two to 3 days and can goes up to weeks for rare serotypes where different media and antisera are necessary to determine the flagella phases (H1 and H2). The drawback is that the current database is limited to the most common serotypes. However, the open design of the CTS method allowing easy addition of new genomic markers and constant expansion of the database continues to address this limitation. SeqSero provided good performance especially with the alpha test version SeqSero 2 and using raw reads. However, as for any *in silico* platform, SeqSero requires an infrastructure and bioinformatics expertise that are currently not available in every laboratory. Cost of sequencing remains one of the most challenging consideration although it has decreased over the years. There is no additional cost associated with SeqSero as the platform is freely available on internet. However, in order to be cost efficient, SeqSero must be considered as a WGS “full package” solution, which includes pathogen characterization, pathogen source tracking and other research applications such as virulence and antibiotic resistance genes determination. Indeed, *in silico* platforms using WGS (e.g., SISTR) have surfaced and each has its advantages and drawbacks but all these technologies will provide detailed genetic analysis of the tested strain (Asthon et al., 2016; Yachison et al., 2017). WGS data gives deeper strain characterization and its high discriminatory power allows inferring close relatedness between isolates. In certain situations, phenotype serotypes that are divergent by antigenic formula are actually close related when analyzing the phylogenetic clusters. In a case study, we tested a set of isolates that were identified either as *S*. Kentucky or a monophasic variant of *S*. Kentucky 8,20:I:- by the traditional phenotypic method. When tested with SeqSero, the method consistently gave *S*. Kentucky as the results. While re-testing the isolates with the traditional phenotypic method, some isolates previously identified as *S*. Kentucky changed to the monophasic variant and vice versa highlighting the versatility of certain serotype (unpublished data).

## Conclusion

Currently, *Salmonella* serotyping remains the first-line characterization for subtyping. However, routine use of the traditional phenotypic method is not always feasible. Moving to alternative molecular methods that are easier to use is a pragmatic approach. Our study indicates that a commercialized solution (CTS assay) provides satisfactory performance when compared to the traditional phenotypic method. Because of its robustness and straightforward utilization, the CTS assay can be easily implemented. Its drawback is the limited database, but this is addressed by continuous updates. In addition, it is possible to build an internal database by correlating a genovar code to a serotype identified by the traditional method. For these reasons, the CTS assay can be a good alternative for routine laboratories. WGS is increasingly used and *Salmonella* serotyping can be performed with WGS data by *in silico* platform, avoiding the use of multiple methods. Our study indicates a satisfactory performance of the *in silico* platform (SeqSero), which can predict more than 2200 serotypes, and provides insight on genetic determinants of serotypes. Nevertheless, routine implementation of this tool relies on systematic sequencing of *Salmonella* isolates. This is the case for public health laboratories where WGS is a common practice and many have transitioned to *in silico* methods.

## Data Availability Statement

All datasets generated for this study have been submitted to the NCBI in BioProject: PRJNA420913 and all SRR numbers are available in the Supplementary Material.

## Author Contributions

BD conceived the study, performed the experiments, analyzed the results, and wrote the manuscript. CB and A-CP performed the bioinformtics analysis, wrote the section Materials and Methods, and reviewed the manuscript. CF performed the sequencing and reviewed the manuscript. AKa and GV performed the CTS analysis and reviewed the manuscript. SL and XD performed the SeqSero interpretation and reviewed the manuscript. AKl reviewed the manuscript and provided approval for publication of the content.

## Conflict of Interest

BD, CB, A-CP, CF, and AKl were employed by the company Nestlé Research. AKa and GV were employed by the company Check-Points. The remaining authors declare that the research was conducted in the absence of any commercial or financial relationship that could be construed as a potential conflict of interest.

## Footnotes

1 http://www.who.int/mediacentre/factsheets/fs139/en/

2 http://www.efsa.europa.eu/en/topics/topic/salmonella

3 www.eurlsalmonella.eu/proficiency-testing/typing-studies

4 http://www.bioinformatics.babraham.ac.uk/projects/fastqc/

5 https://github.com/denglab/SeqSero

6 https://github.com/denglab/SeqSero2

## Abbreviations

AOAC: association of analytical communities
BHI: brain heart infusion
CDC: center for disease control and prevention
CTS: check and trace *Salmonella* assay
DNA: deoxyribonucleic acid
EC: European commission
EFSA: European food safety authority
EU: European union
EURL: European union reference laboratory
FDA: food & drug administration
ISO: international organization for standardization
ISO/TR: international organization for standardization/technical reports
LDR: ligation detection reaction
NCBI: national center for biotechnology information
NGS: next generation sequencing
OIE: world organization for animal health
PCR: polymerase chain reaction
PIR: pathogen isolate repository
RCS: refillable cartridge sets
RIVM: national institute for public health and the environment
SISTR: *Salmonella in silico* typing resource
SKESA: strategic k-mer extension for scrupulous assemblies
SRR: sequence read run
TSA: tryptone soya agar
US: United States
USD: United States dollar
WGS: whole genome sequencing
WKL: white-kauffmann-le minor
XLD: xylose-lysine-désoxycholate
