# Supplementary material for: Salmonella Serotyping; Comparison of the Traditional Method to a Microarray-Based Method and an in silico Platform Using Whole Genome Sequencing Data
Source: Front Microbiol. 2019 Nov 11;10:2554. doi: 10.3389/fmicb.2019.02554 (PMC6859910; doi:10.3389/fmicb.2019.02554)
Supplement: Supplementary file 2 [file Table_2.docx]

**Supplementary Table 2: SeqSero discrepant results**

|  |  | **SeqSero 1** | | **SeqSero 2** | | |
| --- | --- | --- | --- | --- | --- | --- |
| **Strain number** | **Traditional  phenotypic method** | **Raw reads** | **Assembly** | **Raw reads allele  microassembly** | **Raw reads kmer** | **Assembly kmer** |
| PIR02239 | Tennessee | II 6,7:z29:[z42]  or Tennessee | II 6,7:z29:[z42]  or Tennessee | Tennessee | Tennessee | Tennessee* |
| PIR02240 | Muenchen | Virginia  or Muenchen | Virginia  or Muenchen | Muenchen | Muenchen | Muenchen |
| PIR02241 | Goldcoast | Goldcoast  or Brikama | Goldcoast  or Brikama | Goldcoast  or Brikama | Goldcoast  or Brikama | Goldcoast  or Brikama |
| PIR02243 | Tennessee | II 6,7:z29:[z42]  or Tennessee | II 6,7:z29:[z42]  or Tennessee | Tennessee | Tennessee | Tennessee |
| PIR02246 | Poona | Farmsen  or Poona | Farmsen  or Poona | Poona | Poona | Poona |
| PIR02247 | Kiambu | II [1],4,12,27:z:1,5 or Kiambu | II [1],4,12,27:z:1,5 or Kiambu | Kiambu | Kiambu | Kiambu |
| PIR02248 | Muenchen | Virginia  or Muenchen | Virginia  or Muenchen | Muenchen | Muenchen | Muenchen |
| PIR02250 | Goldcoast | Goldcoast  or Brikama | Goldcoast  or Brikama | Goldcoast  or Brikama | Goldcoast  or Brikama | Goldcoast  or Brikama |
| PIR02256 | Oranienburg | II 6,7:m,t:- or Oranienburg | II 6,7:m,t:- or Oranienburg | Oranienburg | Oranienburg | Oranienburg |
| PIR02257 | Molade | Molade  or Wippra | Molade  or Wippra | Molade  or Wippra | Molade  or Wippra | Molade  or Wippra |
| PIR02261 | Ouakam | N/A | Ouakam | N/A | N/A | N/A |
| PIR02263 | Ruiru | Ruiru | N/A | Ruiru | Ruiru | Ruiru |
| PIR02264 | Albany | Albany  or Duesseldorf | Albany  or Duesseldorf | Albany  or Duesseldorf | Albany  or Duesseldorf | Albany  or Duesseldorf |
| PIR02267 | Oranienburg | II 6,7:m,t:- Oranienburg or | II 6,7:m,t:- Oranienburg or | Oranienburg | Oranienburg | Oranienburg |
| PIR02268 | Poona | Farmsen  or Poona | Farmsen  or Poona | Poona | Poona | Poona |
| PIR02271 | Indiana | II 4,12:z:1,7 or Indiana | II 4,12:z:1,7 or Indiana | Indiana | Indiana | Indiana |
| PIR02275 | Senftenberg | Senftenberg  or Dessau | Senftenberg  or Dessau | Senftenberg  or Dessau | Senftenberg  or Dessau | Senftenberg  or Dessau |
| PIR02277 | Oranienburg | II 6,7:m,t:- or Oranienburg | II 6,7:m,t:- or Oranienburg | Oranienburg | Oranienburg | Oranienburg |
| PIR02278 | Tennessee | II 6,7:z29:[z42]  or Tennessee | II 6,7:z29:[z42]  or Tennessee | Tennessee | Tennessee | Tennessee |
| PIR02281 | Indiana | II 4,12:z:1,7 or Indiana | II 4,12:z:1,7 or Indiana | Indiana | Indiana | Indiana |
| PIR02283 | Albany | Albany  or Duesseldorf | Albany  or Duesseldorf | Albany  or Duesseldorf | Albany  or Duesseldorf | Albany  or Duesseldorf |
| PIR02284 | Bracknell | Oudwijk  or Bracknell | Oudwijk  or Bracknell | Bracknell | Bracknell | Bracknell |
| PIR02285 | Senftenberg | Senftenberg  or Dessau | Senftenberg  or Dessau | Senftenberg  or Dessau | Senftenberg  or Dessau | Senftenberg  or Dessau |
| PIR02287 | Mbandaka | Mbandaka | N/A | Mbandaka | Mbandaka | Mbandaka |
| PIR02292 | Hadar | Hadar  or Istanbul | Hadar  or Istanbul | Hadar | Hadar | Hadar |
| PIR02294 | Thompson | Thompson | Ahuza | Thompson | Thompson | Thompson* |
| PIR02295 | Lagos | Tsevie | Lagos | Lagos | Lagos | Lagos |
| PIR02296 | Indiana | II 4,12:z:1,7 or Indiana | II 4,12:z:1,7 or Indiana | Indiana | Indiana | Indiana |
| PIR02297 | Bracknell | Oudwijk  or Bracknell | Oudwijk  or Bracknell | Bracknell | Bracknell | Bracknell |
| PIR02299 | Molade | Molade  or Wippra | Molade  or Wippra | Molade  or Wippra | Molade  or Wippra | Molade  or Wippra |
| PIR02303 | Choleraesuis | Paratyphi C  or Choleraesuis  or Typhisuis | Hissar | Paratyphi C or Choleraesuis or Typhisuis | Paratyphi C or Choleraesuis or Typhisuis | Paratyphi C or Choleraesuis or Typhisuis |
| PIR02310 | Hadar | Hadar  or Istanbul | Hadar  or Istanbul | Hadar | Hadar | Hadar |
| PIR02311 | Poona | Farmsen  or Poona | Farmsen  or Poona | Poona | Poona | Poona |
| PIR02315 | Virchow | Virchow | N/A | Virchow | Virchow | Virchow |
| PIR02317 | Panama | Panama  or Houston | Panama  or Houston | Panama  or Houston | Panama  or Houston | Panama  or Houston |
| PIR02322 | Albany | Albany  or Duesseldorf | Albany  or Duesseldorf | Albany  or Duesseldorf | Albany  or Duesseldorf | Albany  or Duesseldorf |
| PIR02323 | Bracknell | Oudwijk  or Bracknell | Oudwijk  or Bracknell | Bracknell | Bracknell | Bracknell |
| PIR02324 | Hadar | Hadar  or Istanbul | Hadar  or Istanbul | Hadar | Hadar | Hadar |
| PIR02328 | Senftenberg | Senftenberg  or Dessau | Senftenberg  or Dessau | Senftenberg  or Dessau | Senftenberg  or Dessau | Senftenberg  or Dessau |
| PIR02330 | Enteritidis | Blegdam | Enteritidis | Enteritidis | Enteritidis | Enteritidis |
| PIR02336 | Miami | II 9,12:a:1,5 or Miami or Sendai | II 9,12:a:1,5 or Miami or Sendai | Miami  or Sendai | Miami  or Sendai | Miami  or Sendai |
| PIR02337 | 1,4,5,12:i:- | Typhimurium | Potential monophasic  of Typhimurium | Typhimurium | Typhimurium | Typhimurium |
